# Deregulation of KSHV latency conformation by ER-stress and caspase-dependent RAD21-cleavage

**DOI:** 10.1371/journal.ppat.1006596

**Published:** 2017-08-30

**Authors:** Alessandra De Leo, Horng-Shen Chen, Chih-Chi Andrew Hu, Paul M. Lieberman

**Affiliations:** The Wistar Institute, Philadelphia, PA, United States of America; Tulane Health Sciences Center, UNITED STATES

## Abstract

Kaposi’s sarcoma (KS)-associated herpesvirus (KSHV) is a human gammaherpesvirus recognized as the principal causative agent of KS and primary effusion lymphoma (PEL). KSHV establishes persistent latent infection in B lymphocytes where viral gene expression is restricted, in part, by a cohesin-dependent chromosome conformation. Here, we show that endoplasmic reticulum (ER) stress induces a rapid, caspase-dependent cleavage of cohesin subunit RAD21. ER stress-induced cleavage of RAD21 correlated with a rapid and strong viral lytic transcriptional activation. This effect was observed in several KSHV positive PEL cells, but not in other B-cells or non-B-cell models of KSHV latency. The cleaved-RAD21 does not dissociate from viral genomes, nor disassemble from other components of the cohesin complex. However, RAD21 cleavage correlated with the disruption of the latency genome conformation as revealed by chromosome conformation capture (3C). Ectopic expression of C-terminal RAD21 cleaved form could partially induce KSHV lytic genes transcription in BCBLI cells, suggesting that ER-stress induced RAD21 cleavage was sufficient to induce KSHV reactivation from latency in PEL cells. Taken together our results reveal a novel aspect for control and maintenance of KSHV genome latency conformation mediated by stress-induced RAD21 cleavage. Our studies also suggest that RAD21 cleavage may be a general regulatory mechanism for rapid alteration of cellular chromosome conformation and cohesin-dependent transcription regulation.

## Author summary

Latent infection with Kasposi’s Sarcoma (KS)-Associated Herpesivirus (KSHV) is linked to malignant transformation of the host cell. KSHV associated pleural effusion lymphomas (PEL) are highly sensitive to endoplasmic reticulum (ER) stress due to underlying defects in ER stress response pathways. We show here that ER stress inducers lead to a rapid activation of KSHV lytic transcripts, and that an underlying mechanism is found in the caspase and calpain-dependent proteolytic cleavage of RAD21. RAD21 is a subunit of the cohesin complex that maintains a chromosome conformation that restricts KSHV lytic cycle transcription. ER stress-induced cleavage of RAD21 alters the KSHV chromosome conformation associated with latency, and a locus-specific increase in RNA polymerase II association and activation. These findings provide new insights into the regulation of KSHV latency and its response to ER stress, and may further the development of selective treatments for KSHV associated PEL and related malignancies.

## Introduction

Kaposi’s Sarcoma-associated Herpesvirus (KSHV), also known as Human Herpesvirus 8 (HHV8) is strongly associated Kaposi’s Sarcoma and an aggressive subtype of non-Hodgkin's lymphoma, known as Primary Effusion Lymphoma (PEL) [1–3]. KSHV infection normally results in latent infection of B-lymphocytes, where KSHV establishes long-term virus persistence [4]. In the latent state, the viral genomes are maintained in the host nucleus as non-integrated chromatin-associated, circularized genomes, also known as episomes [5]. During latent infection, KSHV expresses only a few viral genes, including LANA, v-FLIP, v-cyclin and microRNAs to maintain the viral latent state and promote host cell proliferation and survival [6, 7]. Periodic reactivation of lytic cycle transcription is required for infectious virus production and transmission, and is also thought to contribute to viral pathogenesis [8].

Reactivation of KSHV lytic cycle typically requires the re-expression of the viral immediate-early transactivator ORF50/Rta, which is necessary and sufficient to trigger lytic cycle transcription in latently infected cells [9]. Suppression of ORF50 transcription during latency is mediated by several different mechanisms, including epigenetic controls of chromatin structure. Histone modifications surrounding the ORF50 gene are known to be regulated through a bivalent histone modification, involving enrichment of Polycomb repressive complexes and associated histone H3K27 trimethylation (H3K27me3) [10–14]. In addition to histone modifications, previous studies from our lab and others have shown that ORF50 transcription and KSHV reactivation can be regulated by subunits of the cohesin complex, as well as the chromatin organizing factor CTCF [15–19]. Cohesins colocalize with CTCF at several loci across the KSHV genome, including strong enrichment at the regulatory regions controlling both latency and lytic transcripts. At the latency control region, cohesins are enriched at CTCF sites clustered in the first intron of the multi-cistronic transcript encoding LANA-vCyclin-vFLIP (ORF73-72-71). At the lytic control region, CTCF sites are distributed upstream of the ORF50 transcription initiation sites, as well as upstream of the divergently transcribed immediate early genes ORF45, 46, 47, 48, and 49. Importantly, we have shown by Chromosome Conformation Capture (3C) assay that the KSHV latency control region forms a DNA-loop interaction with the lytic control region, mediated in part by the cellular CTCF-cohesin complex [16, 17]. Depletion of cohesin subunits, particularly RAD21, leads to the loss of DNA loop formation and transcription derepression of KSHV lytic transcripts for ORF45-49 and ORF50 [16].

Cohesins are known to form a ring-like structure capable of encircling two-strands of duplex DNA. RAD21 forms a critical and highly regulated component of the cohesin ring. During mitosis of each cell cycle, RAD21 (also referred to as SCC1) is cleaved by separase to release sister chromatid cohesion [20]. RAD21 is also subject to proteolytic cleavage by several other pathways, including those activated by caspases in response to various pro-apoptotic signals [21, 22]. RAD21 can also be cleaved by calpain-1 in response to calcium flux [23]. Cleavage of RAD21 appears to be an early event in the apoptotic pathway resulting in the generation of approximately 64- and 60-kDa cleavage products. The apoptotic cleavage site at D279/S of RAD21 is distinct from the previously described mitotic cleavage sites. The specific proteases that cleave RAD21 during apoptosis still need to be identified as well as the physiological significance of RAD21 cleavage with respect to cohesin function during apoptosis [22].

Apoptosis has been shown to trigger an alternative replication pathway for KSHV, characterized by the lack of a requirement for the ORF50 protein, accelerated late gene kinetics, and production of virus with decreased infectivity [24]. Apoptotic caspase-3 activation is necessary and sufficient to initiate this alternative KSHV replication program and may be a common mechanism shared by human herpesviruses [25].

Endoplasmic reticulum (ER) stress triggers the cellular unfolded protein response (UPR) in mammalian cells to ensure correct folding and processing of proteins [26]. However, if the ER stress is not alleviated, the prolonged UPR can induce apoptosis through the caspase cascade [27]. The UPR consisting of the PERK, ATF6, and IRE1-XBP1 pathways attenuates the ER stress by reducing protein translation, increasing the ER folding capacity, and enhancing ER associated protein degradation (ERAD) [28]. Under normal conditions, PERK and ATF6 and IRE1 are associated with an ER chaperone, the immunoglobulin-binding protein (BiP), while the ER stress conditions result in dissociation of BiP, leading to their activation to mitigate this stress [29]. In the absence of PERK or other UPR factors, cells experience severe ER stress and are hypersensitive to its effects [30].

Previous studies suggest that the UPR strongly contributes to regulation of KSHV lytic replication [31–37]. It has been proposed that the viral early lytic proteins, including ORF47/45, may control and promote BiP expression to protect host cells from ER stress to facilitate the assembly and release of virions [31]. A recent study shows that KSHV-encoded LANA and v-cyclin can suppress the transcription of the ER stress sensors IRE1 and PERK to sensitize PEL cells to ER stress inducers [32, 33]. Moreover, XBP-1 spliced form, normally absent in PEL cells, is potently induced by ER stress and binds to and activates the KSHV ORF50 promoter, thereby inducing KSHV lytic replication [34]. However, another study has reported that UPR has an inhibitory effect on KSHV replication and gene expression [37]. The precise roles and the effects of ER stress in KSHV reactivation from latency remain to be clarified. Here, we describe the effect of ER stress on RAD21 cleavage in latently infected PEL cells and the corresponding changes in KSHV chromosome architecture and lytic gene transcription.

## Results

### ER stress inducers promote RAD21 cleavage and KSHV lytic gene expression in latently infected PEL cells

**Fig 1:**
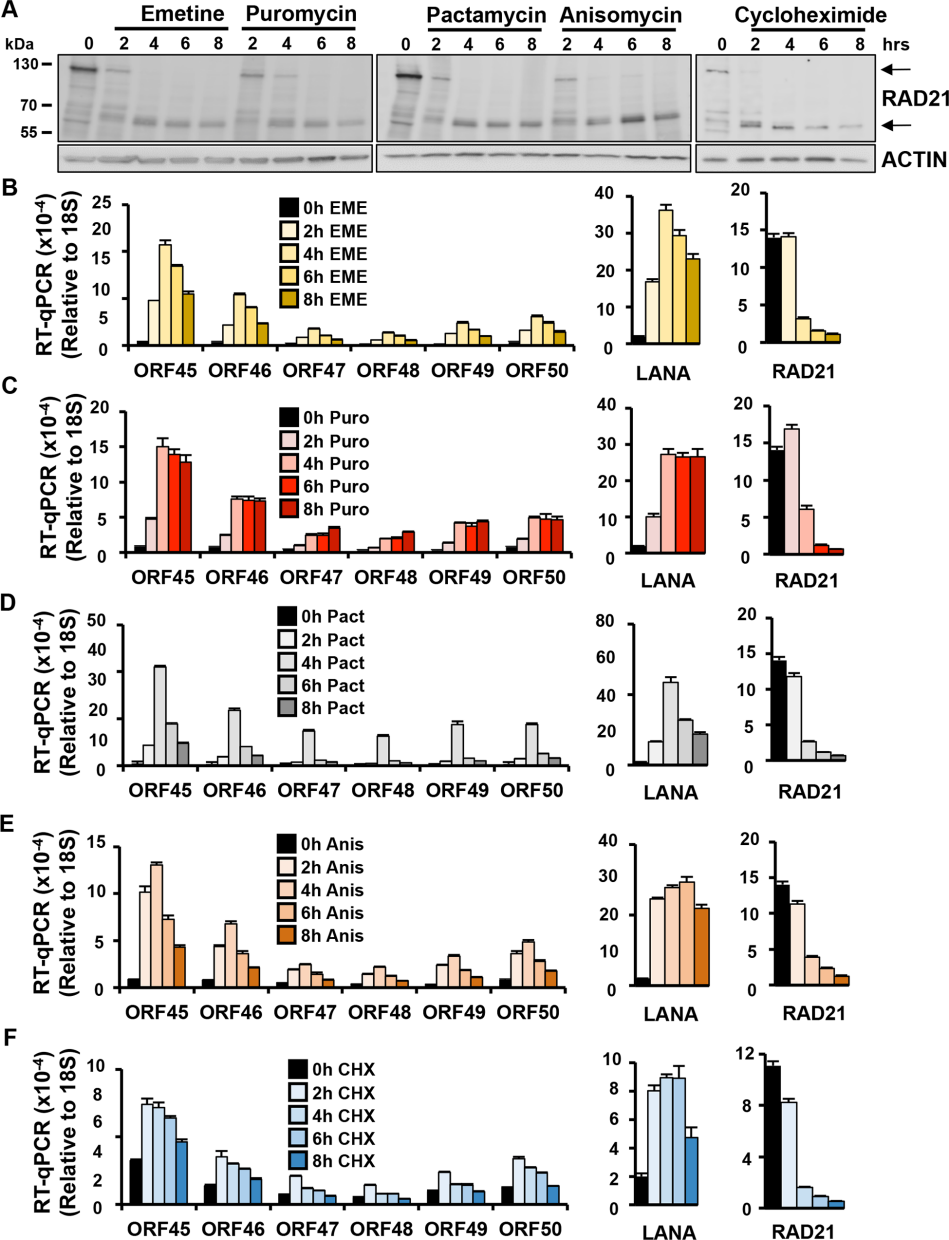
Induction of RAD21 degradation and KSHV lytic cycle by protein synthesis inhibitors in BCBLI cells. **(A)** Immunoblotting of RAD21 in KSHV latently infected BCBLI cells exposed to different antibiotics. BCBLI cells were incubated with 10ug/ml Emetine, 75ug/ml Puromycin, 0.2 ug/ml Pactamycin, 10ug/ml Anisomycin or 100 ug/ml cycloheximide for 0, 2, 4, 6 and 8 hours. **(B-F)** RT-qPCR for ORF45-50, LANA and RAD21 transcripts relative to cellular 18S in BCBLI cells treated for 0–8 hrs with Emetine (EME), Puromycin (Puro), Pactamycin (Pact), Anisomycin (Anis) or Cycloheximide (CHX) as described in (A).

Pharmacological inhibitors of protein translation are potent inducers of ER-stress [27]. To investigate how ER-stress alters RAD21 and KSHV lytic reactivation, we assayed a panel of protein translation inhibitors for their effect on latently infected PEL cells. BCBL1 cells were treated with either emetine, puromycin, pactamycin, anisomycin, or cyclohexamide and then assayed for RAD21 cleavage (Fig 1A), or KSHV lytic gene transcription (Fig 1B–1F). Every protein synthesis inhibitor tested resulted in the rapid (within 4 hrs) and complete cleavage of the ~130 kDa RAD21 protein to generate fragments of ~64- and 60-kDa, as determined by Western blot assay (Fig 1A). RT-PCR analysis revealed KSHV transcripts for lytic immediate early genes ORF45-50, as well as LANA were increased several folds within 2–4 hrs after treatment (Fig 1B–1F). In contrast, cellular transcripts for RAD21 were unaltered at 2 hrs, and reduced by 4 hrs of treatment.

**Fig 2:**
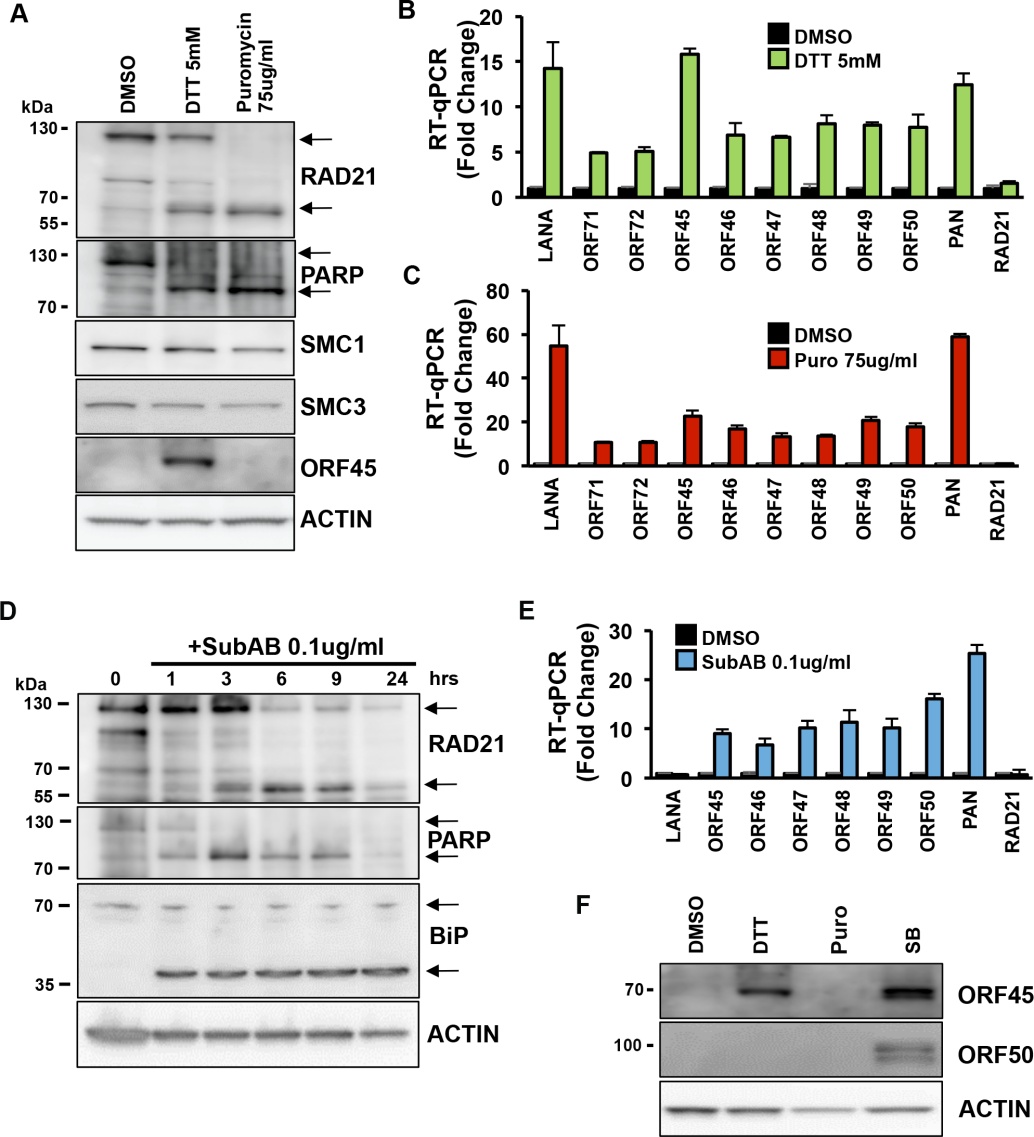
ER stress inducers promote RAD21 cleavage and KSHV lytic reactivation in BCBLI cells. **(A)** Immunoblotting of RAD21, PARP, SMC1, SMC3, ORF45 and actin in BCBLI cells exposed to 5mM DTT or 75ug/ml Puromycin (Puro) for 6 hours. **(B-C)** RT-qPCR for latent transcripts (LANA, ORF71, ORF72), lytic transcripts (ORF45-50, PAN), and RAD21 transcript relative to cellular actin in BCBLI cells treated as in (A). The data are expressed as fold change of the treated versus untreated (DMSO) cells. **(D)** Immunoblotting of RAD21, PARP, BiP and actin in BCBLI cells exposed to 0.1 ug/ml of SubAB for 1, 3, 6, 9 and 24 hours. **(E)** RT-qPCR for latent transcript LANA, lytic transcripts (ORF45-50, PAN), and RAD21 transcript relative to cellular actin in BCBLI cells treated as in (D). The data are expressed as fold change of the treated versus untreated (time 0h) cells. **(F)** Immunoblotting of ORF45, ORF50, and actin in BCBLI cells exposed to 5mM DTT or 75ug/ml Puromycin (Puro) or 3mM sodium butyrate (SB) for 6 hours.

To determine if other agents known to induce ER-stress had similar effects on RAD21 and KSHV transcription, we assayed the reducing agent dithiothreitol (DTT) along with puromycin (Fig 2A–2C). DTT is known to induce ER stress in cultured B-lymphocytes [38]. DTT (5 mM) treatment for 6 hrs resulted in the partial (~50%) cleavage of RAD21 to generate the smaller (60 kDa) form, while puromycin led to a more complete cleavage of RAD21 (Fig 2A). Both DTT and puromycin led to a near complete cleavage of PARP1, suggesting that apoptotic caspases were activated. Other cohesin components, SMC1 and SMC3, responsible for the cohesin ring structure assembly with RAD21 were not greatly affected by DTT or puromycin treatments, suggesting that RAD21 represented the main cleavage target in the cohesin complex during the ER stress-associated apoptosis. We also found that DTT exposure induced the expression of ORF45 viral lytic protein (Fig 2A) and viral lytic genes transcription (Fig 2B). As expected, puromycin induced lytic transcription (Fig 2C), but did not induce ORF45 protein expression (Fig 2A), consistent with its inhibition of protein synthesis. These results were validated in three other PEL cell lines (BC3, BC1 and JSC1), showing similar effects on RAD21 cleavage and viral lytic activation obtained in BCBLI cells after DTT or puromycin treatment (S1 Fig).

We also assayed more biological ER stress inducers. The bacterial-derived subtilase cytotoxin (SubAB) is a potent inducer of ER stress that has been shown to directly cleave the ER-chaperone BiP [39] (Fig 2D and 2E). As expected, SubAB’s direct target, BiP, was cleaved already after 1 hour treatment, while partial cleavage of RAD21 and PARP1 occurred only after 3 hours treatment (Fig 2D). Twenty-four hours after the start of SubAB exposure, lytic genes transcriptional levels were increased 10- to 25-fold compared to time 0h (Fig 2E). We also tested tunicamycin, a natural product inhibitor of glycosylation and well-characterized inducer of the ER stress (S2D and S2E Fig). Similar to DTT, tunicamycin induced a partial cleavage (~25%) of RAD21, and more extensive cleavage of PARP1 (S2D Fig), indicating it is affecting apoptotic caspases. Tunicamycin induced a small increase in ORF50 (~2 fold) and PAN (~5 fold), but reduced the transcripts for LANA, suggesting its effect on KSHV is quantitatively or qualitatively distinct from that of protein synthesis inhibitors. These results indicate that different ER stress inducers partially cleave RAD21 and stimulate KSHV lytic cycle transcription, and that RAD21 cleavage correlates with KSHV reactivation.

Host cell apoptosis can induce an alternative pathway for KSHV lytic reactivation pathway that is independent of ORF50 immediate-early protein expression [24]. To assess the process of reactivation observed under ER stress used in these experiments, we assayed ORF50 and ORF45 protein expression by Western blot after treatment of BCBL1 cells with either DTT, puromycin, or sodium butyrate (SB) (Fig 2F). We found that DTT induced ORF45, but not ORF50 protein. In contrast, SB induced both ORF45 and ORF50, while puromycin produced no viral protein expression, as expected. These results suggest that ER-stress inducers, like DTT, induce KSHV early lytic gene expression in the absence of ORF50 protein, similar to what has been reported for apoptosis inducers [24].

### Inhibition of RAD21 cleavage and KSHV reactivation by caspase peptide inhibitors

**Fig 3:**
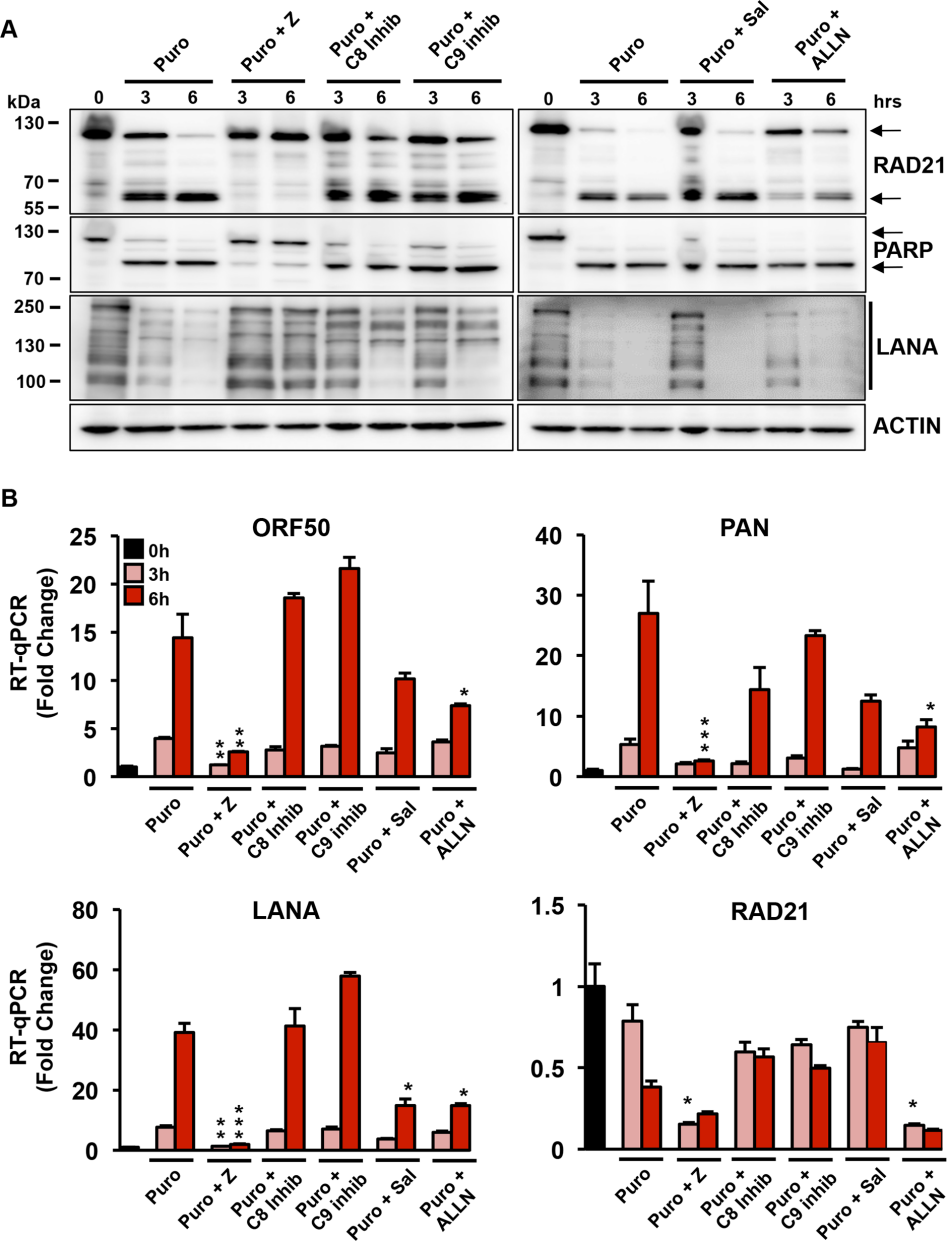
Effects of the caspase inhibitors on RAD21 cleavage and KSHV replication induced by treatment with puromycin in BCBLI cells. **(A)** Western blot analysis for RAD21, PARP, LANA and actin in BCBL1 cells treated for 3 and 6 hrs with Puromycin (Puro) in the presence or in the absence of 20 uM pan-caspase inhibitor Z-VAD-FMK (Puro + Z), 20 uM caspase 8 inhibitor Ac-AEVD-CHO (Puro + C8 inhib), 20 uM caspase 9 inhibitor Ac-LEHD-CHO (Puro + C9 inhib), 20 uM Salubrinal (Puro + Sal) and 50 uM calpain inhibitor I ALLN (Puro + ALLN). **(B)** RT-qPCR for ORF50, PAN, LANA and RAD21 transcripts relative to cellular actin in BCBLI cells treated as in (A). The data are expressed as fold change of the treated versus untreated (0h) cells. The significance is expressed as inhibitors treated versus only puromycin treated cells * p < 0.05, ** p < 0.01, *** p < 0.001.

To gain further insight into the proteases responsible for RAD21 cleavage and KSHV reactivation, we investigated the effects of peptide-based caspase inhibitors on ER stress-induced RAD21 cleavage (Fig 3A) and viral lytic transcriptional activation (Fig 3B). BCBLI cells were treated with 20 uM z-VAD-FMK, a broad-spectrum caspase inhibitor, 30 min prior to puromycin (75 ug/ml) treatment. Treatment with z-VAD-FMK completely blocked puromycin-induced RAD21 cleavage and greatly prevented PARP1 cleavage (Fig 3A and quantified in S3). This event was accompanied by a significant suppression of ORF50, PAN and LANA viral genes transcription (Fig 3B). In contrast, pretreatment of puromycin-induced BCBLI cells with 20 uM caspase 8 and 9 inhibitors or with 20 uM salubrinal (a specific PERK inhibitor) did not significantly affect cleavage of RAD21 or PARP1 nor the activation of KSHV lytic genes. The calpain 1 inhibitor ALLN partially reduced puromycin-induced cleavage of RAD21 but not PARP1, indicating the possible involvement of calpain 1 in the ER stress-mediated pathway cleavage of RAD21. Moreover, ALLN dampened puromycin-induced activation of KSHV lytic genes by ~50%. Taken together, these studies suggest that ER stress-induced RAD21 cleavage leads to reactivation of KSHV early lytic transcripts. These findings also suggest that RAD21 is sensitive to a caspase and calpain-dependent proteolysis.

KSHV LANA is also known to be a substrate for caspases, with cleavage sites in both its N- and C- terminal domains [40]. To assess whether ER stress leads to a caspase-dependent cleavage of LANA, we treated BCBL1 cells with puromycin and various protease inhibitors followed by Western blot analysis (Fig 3A, lower panel). Similar to RAD21, puromycin-treatment led to a rapid loss of LANA protein. The pan-caspase inhibitors z-VAD-FMK prevented LANA cleavage at 3 and 6 hrs, while caspase 8 and 9 inhibitors and the ER stress inhibitor salubrinal reduced LANA cleavage at 3 hours, but had no measurable effect at 6hrs. The calpain 1 inhibitor ALLN did not affect LANA cleavage indicating that calpain proteases are not involved in this event. These results suggest that LANA is also subject to rapid caspase cleavage induced by ER-stress, and this may also contribute to deregulation of KSHV latency.

### ER stress induces RAD21 cleavage and KSHV reactivation in PEL cells

**Fig 4:**
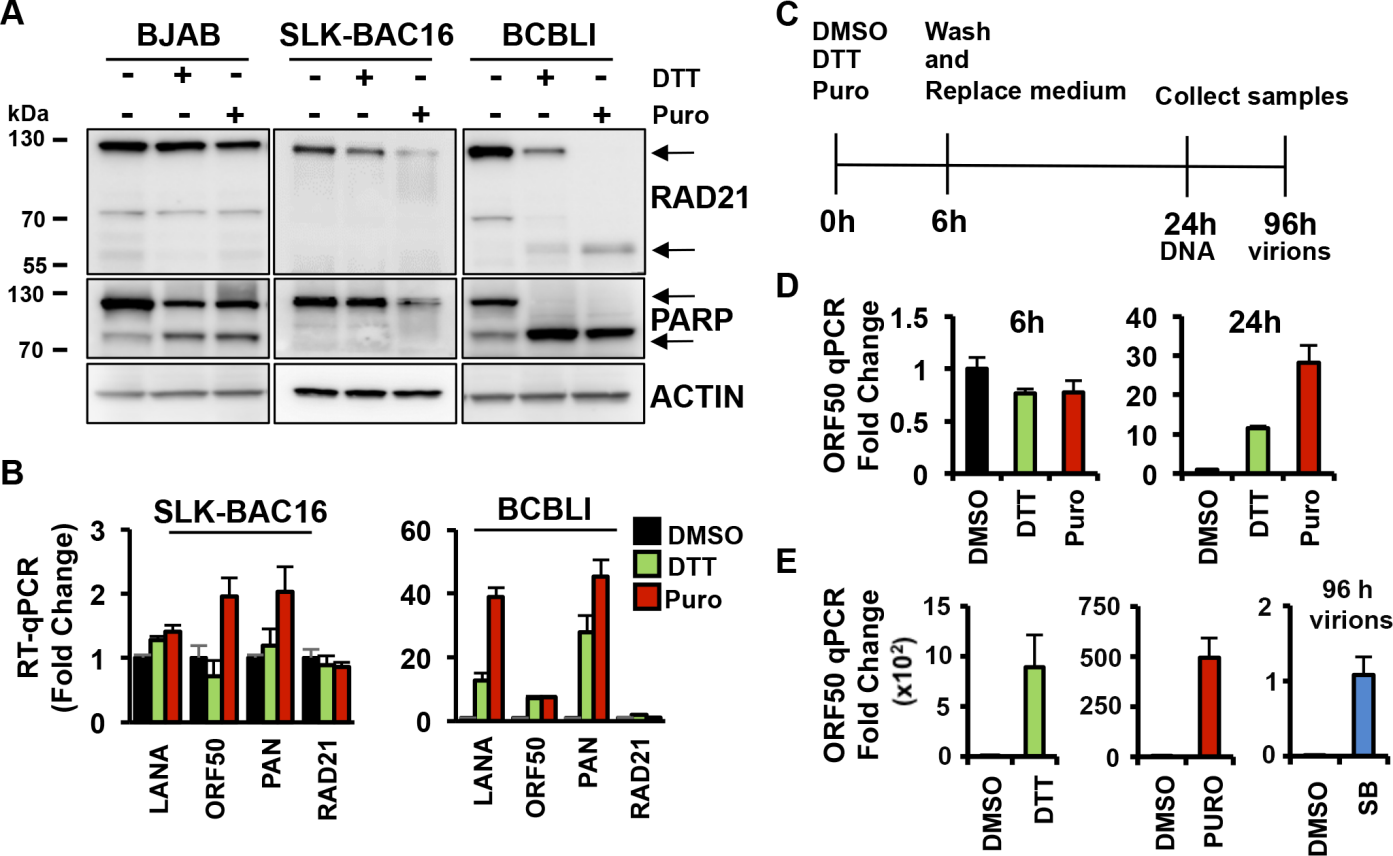
ER stress induces KSHV lytic replication in BCBL1 cells. **(A)** Immunoblotting of RAD21, PARP and actin in BJAB, SLK-BAC16 and BCBL1 cells exposed to 5mM DTT or 75ug/ml Puromycin (Puro) for 6 hours. **(B)** RT-qPCR for latent transcript LANA, lytic transcripts ORF50 and PAN, and RAD21 transcript relative to cellular actin in SLK-BAC16 and BCBL1 cells treated with DMSO, 5mM DTT or 75ug/ml Puromycin (Puro) for 6 hours. The data are expressed as fold change of the treated versus untreated (DMSO) cells. **(C)** Schematic representation of drug treatments in BCBL1 cells. Cells were treated with a 6-hour pulse of DMSO or 5mM DTT or 75ug/ml Puromycin (Puro) and then cultured in fresh media lacking drugs for the remaining 24 hours or 96 hours. **(D)** At 6 and 24 hours after drug treatment as described in (C), the level of viral DNA was evaluated by real-time qPCR for ORF50 DNA relative to cellular actin. The data are expressed as fold change of the treated versus untreated (DMSO) cells. **(E)** Virion DNA was isolated from cell supernatants at 96 h post-treatment and wash-out and assayed by qPCR for ORF50 DNA. Sodium butyrate (SB) treated cell supernatant was used as positive control. The data are expressed as fold change of the treated versus untreated (DMSO) cells.

Since PEL cells are reported be more sensitive to ER stress than KSHV-negative lymphoma cells [24], we evaluated the effects of 6-hour exposure to DTT and puromycin on RAD21 cleavage in several other cell types, including KSHV-negative BJAB cells and *in vitro* infected SLK-BAC16 cells, compared to BCBLI cells (Fig 4A). Both treatments selectively promoted RAD21 cleavage in PEL cells, whereas BJAB and SLK-BAC16 cells were more resistant to ER stress-induced apoptosis at the same time and dose of exposure as also confirmed by PARP1 cleavage (Fig 4A). We next investigated whether SLK-BAC16 cells were as responsive to KSHV lytic activation as BCBLI cells by RT-qPCR (Fig 4B). Six-hour treatment with puromycin or DTT had no significant effects on KSHV reactivation in SLK-BAC16 cells. These findings confirm that PEL cells are more sensitive to ER stress and this sensitivity correlates with induction of KSHV lytic reactivation.

To determine whether ER stress-induced activation of KSHV early lytic gene transcripts could lead to the full activation of KSHV lytic DNA replication, we monitored the levels of KSHV DNA in BCBLI cells treated transiently with DTT and puromycin (Fig 4C). We pulse-treated BCLB1 cells with DTT or puromycin for 6 hours, and then washed cells to avoid an irreversible apoptotic response. Remarkably, at 24 hours after the 6-hour pulse treatment, intracellular KSHV DNA levels increased more than 10 fold for DTT treatment and about 30 fold to puromycin treatment, demonstrating that puromycin- and DTT-induced reactivation leads to lytic virus amplification in BCBLI cells (Fig 4D). We also measured viral DNA isolated from virions in the cell supernatants at 96 hrs after treatment and recovery. We found that DTT and puromycin lead to a 10 and 500 fold increase, respectively, in virion DNA relative to sodium butyrate treatment (Fig 4E). Thus, ER-stress inducers can trigger the complete KSHV lytic replication cycle and virion production.

### Epigenomic programming of KSHV latent and lytic regulatory regions during ER-stress associated apoptosis

**Fig 5:**
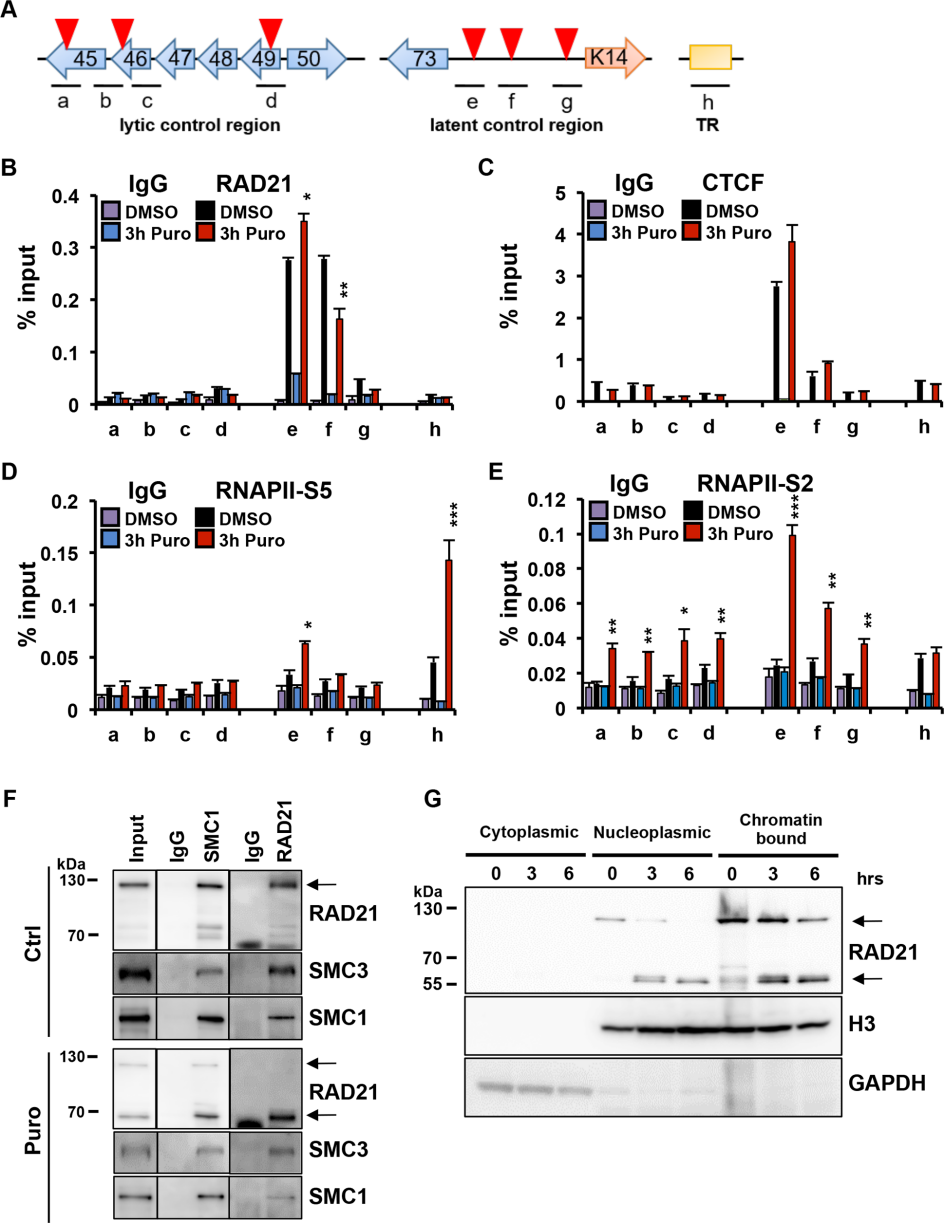
Effect of ER stress inducers on the epigenomic programming of KSHV regulatory control regions. **(A)** Schematic of KSHV lytic control region, latency control region and terminal repeats region (TR) with primer positions a-h used for ChIP assays. Red triangles represent CTCF binding sites. **(B-E)** BCBL1 cells treated for 3 hrs with DMSO or 75 ug/ml Puro were assayed by ChIP for IgG or **(B**) RAD21, **(C)** CTCF, **(D)** RNA Polymerase II phosphoS5 (RNAPII-S5) and **(E)** RNA Polymerase II phosphoS2 (RNAPII-S2). **(F)** BCBL1 cells were treated with puromycin for 6 hr, processed for IP with antibody to IgG, SMC1 or RAD21 and then assayed by Western blot with antibody to RAD21, SMC3 or SMC1. **(G)** BCBL1 cells were treated with puromycin for 3 and 6 hr, subcellular fractionation was performed, and cytoplasmic, nucleoplasmic and chromatin fractions were analyzed by Western blotting. GAPDH (a cytoplasmic protein), and chromatin bound histone H3 protein were used as controls for subcellular fractionation.

To investigate changes in the KSHV chromatin in response to the ER-stress, we use chromatin-immunoprecipitation (ChIP) with several different antibodies and genomic locations of regulatory significance (Fig 5A). We first performed ChIP assays for RAD21 and CTCF after 3-hour puromycin treatment, a time that is likely to avoid potential complications due to apoptotic chromatin condensation (Fig 5B and 5C). Puromycin treatment had a small, disparate effect on RAD21 binding at the latency control region (primers e-g), with small increase at primer position e (overlapping CTCF binding sites), but a reduction at primer position f (overlapping the LANA transcription start site) (Fig 5A). RAD21 ChIP did not change at the lytic control region (primers a-d) or the TR (primer h) (Fig 5B). CTCF binding to the KSHV genome was not significantly affected by the puromycin treatment (Fig 5C). In contrast, RNA pol II phosphoisoform S2 (RNAPII-S2), associated with transcription elongation, was enriched within the latency control region and the lytic control region, while there was no significant enrichment at the TR compared to control at early time points (3 hours) of exposure (Fig 5E). RNA pol II phosphoisoform S5 (RNAPII-S5), associated with transcription initiation and pausing, was found most enriched at the TR region (primer position h) and the CTCF site in the LANA intron (primer position e) (Fig 5D). RNAPII-S5 and -S2 were also examined at additional regions throughout the lytic immediate early transcription region, where they were found to be enriched across the entire region after puromycin treatment (S4 Fig).

To determine if ER stress induction affected cohesin complex assembly through RAD21 cleavage, we assayed the effects of 6-hour pulse treatment with puromycin by coimmunoprecipitating RAD21 with SMC1 or SMC3 (Fig 5F). We found that the treatment did not disrupt the interaction of RAD21 with SMC1 and SMC3, indicating that cleaved RAD21 fragments were still associated with other cohesin components. The caspase-cleaved form of RAD21 was also found to remain associated with the chromatin and soluble nuclear fractions, and not released to the cytoplasm (Fig 5G). Collectively, these results indicate that RAD21 cleavage did not cause the complete disassembly of the remaining cohesion components, nor its dissociation with chromatin and the KSHV genome.

### ER stress-induced RAD21 cleavage causes a disruption of DNA linkage between KSHV latent and lytic control regions

**Fig 6:**
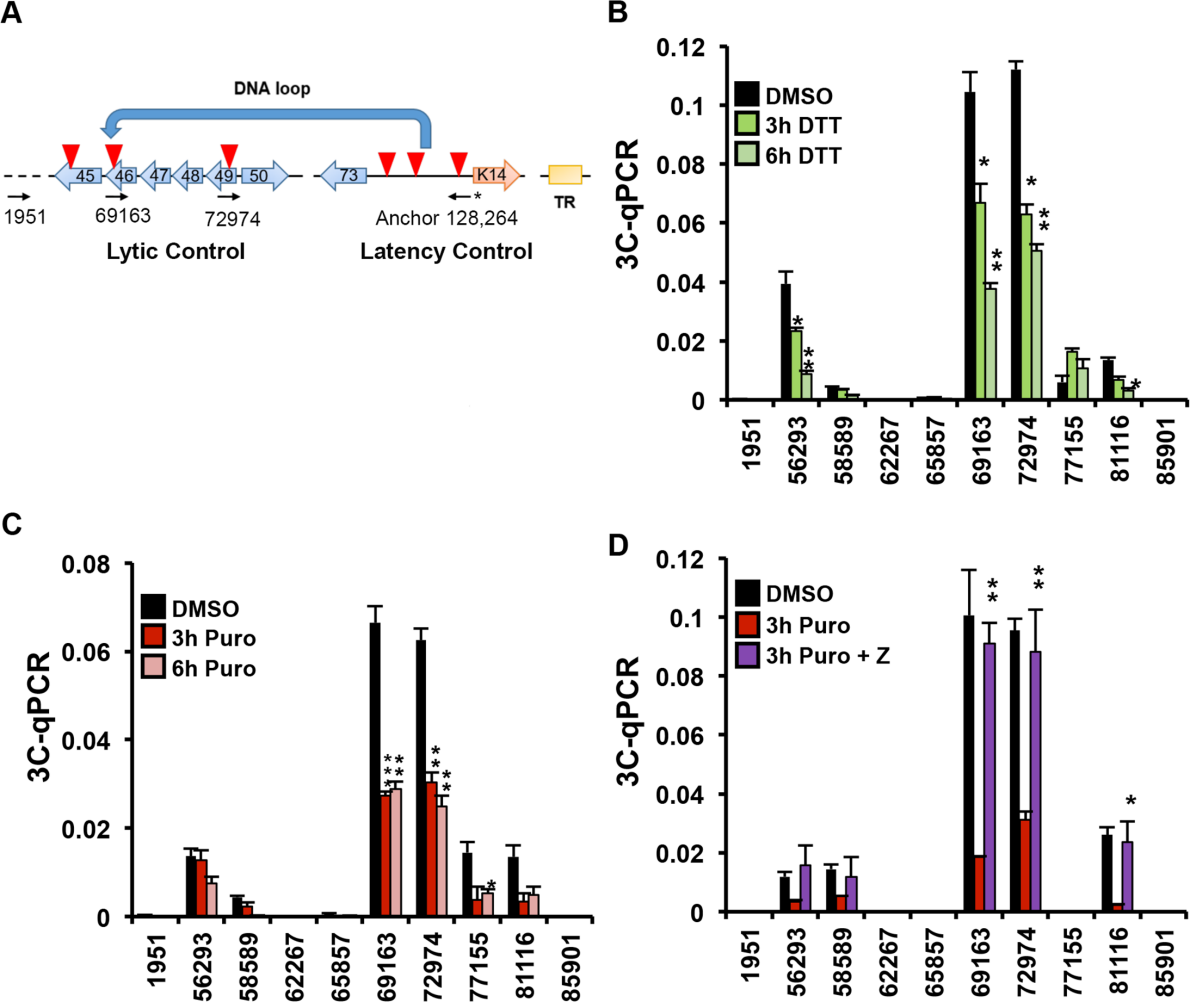
RAD21 cleavage promotes DNA-loop disruption between KSHV latent and lytic control regions. **(A)** Schematic of KSHV regulatory regions and primers used for 3C experiment. Anchor primer at latency control region (128,264) indicated by asterisk (*) and acceptor sites in lytic control region (69163), and others regions are indicated. Red triangles represent CTCF binding sites. **(B-C)** Chromosome conformation capture **(**3C) assay in BCBL1 cells treated for 3–6 hrs with DMSO or 5mM DTT (B) or 75 ug/ml Puro (C) using anchor and acceptor primers, as indicated in (A). **(D)** Chromosome conformation capture **(**3C) assay in BCBL1 cells treated for 3 hrs with 75 ug/ml Puro in the presence or in the absence of 20 uM z-VAD-FMK using anchor and acceptor primers, as indicated in (A). * p < 0.05, ** p < 0.01, *** p < 0.001.

It has been previously shown the presence of a DNA-loop between the latent and lytic control regions of KSHV in PEL cells [17], and depletion of RAD21 leads to a disruption of this loop favoring KSHV reactivation from latency [16]. To determine whether RAD21 cleavage might be responsible for the loss of these viral DNA interactions, we performed Chromatin Conformation Capture (3C) assays with an anchor primer at the KSHV latency control region (Fig 6A). As previously reported [17], we detected a selective interaction between the latent control region and the lytic control region (primer 69163 and 72974) in BCBLI cells (Fig 6B and 6C). Treatment with DTT or puromycin for 3 or 6 hours reduced major 3C interactions at lytic control region by ~60%, as well as other weaker 3C interactions at positions downstream (56293) and upstream (77155 and 81116) of the lytic control region. To further investigate the caspase-dependence on the disruption of the KSHV DNA loop between the lytic and the latency control region, we repeated the 3C assay following puromycin treatment in the presence of the pan-caspase inhibitor z-VAD-FMK (Fig 6D). Treatment with the caspase inhibitor reversed the disruptive effect of puromycin and maintained the latency DNA conformation. These findings suggest that ER-stress associated caspase cleavage of RAD21 alters KSHV latency through disruption of the KSHV DNA conformation.

### C-terminal RAD21 cleavage product promotes KSHV reactivation

**Fig 7:**
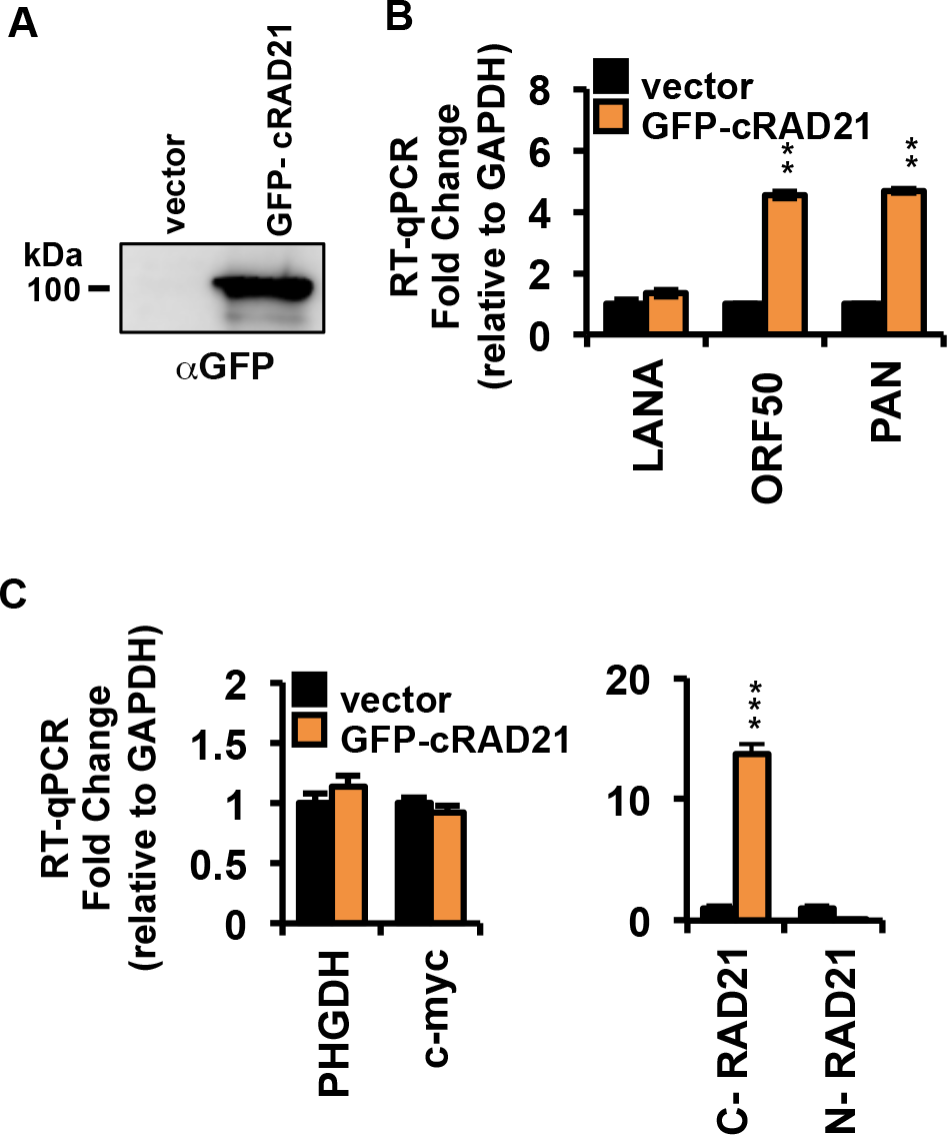
Effect of the expression of RAD21 C- terminal (aa 280–631) on KSHV reactivation and apoptosis. **(A)** BCBL1 cells were transiently transfected with the GFP-tagged mammalian expression vector encoding the cleavage product hRAD21 C-terminal (aa 280 to 631). Empty vector used as a control. At 72 h post-transfection, cells lysates were subjected to immunoprecipitation with IgG or anti-GFP antibody followed by Western blot analysis with GFP antibody. **(B)** BCBL1 cells treated as in (A) were subjected to RNA extraction and RT-qPCR for latent transcript LANA, lytic transcripts ORF50 and PAN. (C) Same as in panel B, except RT-qPCR for cellular genes PHGDH and myc tested as cellular negative controls (left panel), or the 3’ end of RAD21 (ectopic transcript), or the 5’ end of RAD21 (endogenous transcript). * p < 0.05, ** p < 0.01, *** p < 0.001.

It has been reported that the C-terminal RAD21 cleavage product has pro-apoptotic activity [21, 22] We tested whether the overexpression of the RAD21 C-terminal cleavage product was sufficient to trigger KSHV reactivation in BCBLI cells. The experiment was performed by transient transfection of BCBLI cells with cytomegalovirus (CMV) promoter-driven GFP-tagged mammalian expression plasmid encoding the cleavage product, RAD21 C-terminal (aa 280 to 631) protein, or with the empty vector. After 72 hours, cells were sorted for GFP and the expression of the small RAD21 fragment was analyzed by SDS-PAGE (Fig 7A). Evaluation of KSHV lytic induction was obtained by RT-qPCR measuring the transcriptional levels of ORF50 and PAN genes (Fig 7B). Cells transfected with C-terminal RAD21 plasmids indicated a significant increase of about 4–5 fold of ORF50 and PAN transcripts compared with those in cells transfected with vector control (Fig 7B). In contrast, latent LANA transcripts were not significantly altered by the presence of RAD21 C-terminal fragments. Overexpression of C-terminal RAD21 cleavage product did not affected the transcription of the cellular genes PHGDH and c-myc tested as negative controls (Fig 7C, left), nor did it alter endogenous RAD21 expression (Fig 7C, right), as determined using two different primer sets for the transcripts encoding the N and C-terminal domains of RAD21 (Fig 7C). These findings indicate that the C-terminal RAD21 cleavage product is sufficient to trigger a partial reactivation of KSHV lytic gene transcription in BCBL1 cells.

## Discussion

RAD21 has been implicated in the control of KSHV latency since its depletion leads to the reactivation of lytic cycle gene expression from latently infected PEL cells [16, 17]. In this study, we reported the impact of ER stress-associated RAD21 cleavage in the regulation of KSHV latent to lytic switch in PEL cells. Our data indicate that the induction of ER stress by different stimuli is sufficient to trigger caspase-mediated RAD21 cleavage associated with a rapid activation of KSHV lytic transcription. We found that some inducers of ER stress, such as protein synthesis inhibitors and DTT, were more robust and rapid in inducing RAD21 cleavage and lytic reactivation, while other ER stress inducers, such as tunicamycin and SubAB cytotoxin, induced only partial cleavage of RAD21 with slower kinetics and lower amplitude of KSHV lytic reactivation. The slower kinetics of SubAB and tunicamycin-induced reactivation of KSHV may reflect the fact that they cause a distinct ER stress compared to more broadly acting chemical inhibitors [41]. It is also likely that protein-synthesis inhibitors and reducing agents like DTT affect additional pathways and activate a broader range of caspases and calpains, which result in more complete cleavage of RAD21 and other regulatory targets. The correlation between the extent of RAD21 cleavage and the amplitude of KSHV reactivation supports the model that RAD21 is one of several critical restriction factors for KSHV latent-to-lytic switch.

In contrast to our findings, a previous study found that ER stress alone does not cause ORF50 expression and lytic replication in latently infected PEL cells, but only enhances lytic gene expression and viral production in otherwise reactivated PEL cells [6]. However, this study focused on the effects of thapsigargin, which like tunicamycin, is likely to have a slower and weaker effect on RAD21 cleavage than protein synthesis inhibitors or DTT that were the focus of experiments presented here. Perhaps more consistent with our findings, is a previous report demonstrating an alternative, ORF50-independent reactivation pathway triggered by apoptosis that requires initiation of caspase activity [24]. Our findings that ER-stress inducers stimulate a rapid activation of transcription in the absence of new protein synthesis are also consistent with an ORF50-independent activation of immediate early and early-gene transcription.

Multiple factors that function upstream and independent of ORF50 to initiate the KSHV reactivation from latency have been described [42, 43]. HIF1-responsive hypoxia [44, 45], Notch1 signaling [46], and HDAC inhibition [47, 48] are all known to function at the ORF50 promoter locus to initiate transcription in latently infected PEL cells. Whether ER stress-inducers function through one of these pathways is not yet known. It is also possible that protein-synthesis inhibitors may prevent the expression of an unstable repressor. We suggest that one such repressor of KSHV lytic cycle is RAD21. In this case, the rapid turnover is facilitated by ER stress-induced caspase cleavage.

The mechanism of RAD21 cleavage may be different than that of other apoptotic targets. By using different protease inhibitors, we found that only the pan-caspase inhibitor ZVAD and the calpain-inhibitor ALLN greatly decreased the levels of RAD21 cleaved product, and reduced the expression of KSHV lytic genes. The calpain-inhibitor ALLN partially prevented RAD21 cleavage, but did not have any effect on PARP1, suggesting a specific role of the calcium-dependent calpain pathway in regulating RAD21 cleavage and KSHV reactivation. This also suggests that RAD21 cleavage, and not loss of PARP1 or other caspase targets, is necessary for transcription reactivation of KSHV. The C-terminal product of RAD21 has been shown to have pro-apoptotic activity [21, 49]. We found that ectopic expression of the C-terminal RAD21 fragment is sufficient to induce KSHV reactivation from latency in BCBL1 cells (Fig 7). This suggests that RAD21 cleavage and the RAD21 cleavage product are active inducers of KSHV immediate early gene transcription.

Induction of ER stress with clinically approved drugs like bortezomib has been shown to be an effective treatment of certain subtypes of cancers [50]. Recently, lenalidomide has also been reported to induce progressive ER stress in multiple myeloma cells [51] and to have favorable results in treatment of PEL patients [52]. PEL cells have been found to be highly sensitive to ER stress and ER stress-inducing agents, like chloroquine, have been shown to selectively inhibit PEL cell growth [53]. We found that ER stress induced RAD21 cleavage in PEL cells, but not SLK-BAC16 cells or KSHV negative B-lymphoma cells, such as BJAB. The particular sensitivity of PEL cells to ER stress has been reported previously, and shown to be linked to defects in the ER stress sensor IRE1α and PERK [32]. The ER stress sensitivity of PEL cells may also be significant in determining the epigenetic mechanisms controlling KSHV latency, as well as its particular sensitivity to RAD21 degradation or depletion.

The response to ER stress in PEL cells can be observed in viral chromatin structure at relatively early time points. ChIP experiments revealed that RNAPII accumulates throughout the entire immediate early gene locus spanning the ORF45-ORF50 transcripts, and was found to be highly enriched at several other locations including the TR (Fig 5). These findings suggest that ER stress induces a rapid reprogramming of RNAPII and a global access to latent viral genomes. RNAPII can be generally activated in response to several stress inducers, such as nucleotide depletion [54] or the chemical HMBA [55, 56], that liberate the transcription elongation factor pTEFb to promote transcription of target genes, such as latent HIV. ER stress degradation of RAD21 may function in a similar manner to release a repressed or paused RNA polymerase II at the KSHV lytic control locus.

**Fig 8:**
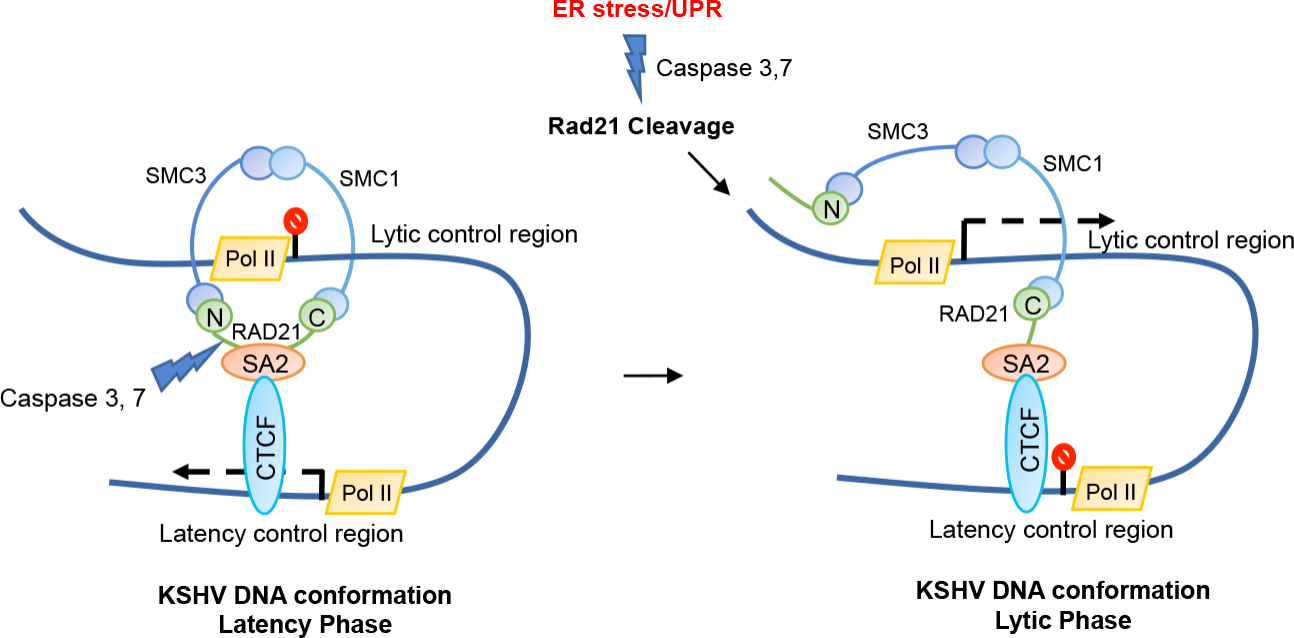
Model of RAD21 cleavage and KSHV lytic induction by ER stress inducers. ER stress leads to activation of the UPR pathway and apoptotic caspase cascade. Apoptosis triggers the degradation of cellular RAD21, one of the main proteins responsible for the maintenance of KSHV episome conformation during latency. The cleavage of RAD21 promotes the disruption of the KSHV DNA loop between the latency and lytic regulatory control regions and consequently the switch to KSHV lytic phase.

An additional insight into the role of RAD21 regulating KSHV reactivation may be provided by an examination the properties of cleaved RAD21. ChIP analysis of RAD21 binding to KSHV genome at early time points (3h) after ER-stress revealed only a minimal loss of RAD21 binding to viral chromatin, despite the fact that most RAD21 protein was cleaved at this time point (Fig 5). IP studies also revealed that cleaved forms of RAD21 could efficiently interact with cohesin subunits SMC1 and SMC3 (Fig 5F). Previous studies showed that the amino- and carboxy-terminal domains of RAD21 interact with the head regions of SMC3 and SMC1, respectively, and the central region of RAD21 binds to SA [57]. In particular, RAD21 binds to SA proteins through two SA-binding motifs on RAD21, located at 60–81 aa of the N-terminal and 383–392 aa of the C-terminal of RAD21, respectively, and therefore not affected by caspase-dependent cleavage of RAD21 [58]. Moreover, the interaction of CTCF with the cohesin complex involves only direct contacts between the cohesin subunit SA2 and the C-terminal tail of CTCF [59]. These findings suggest that ER stress-induced RAD21 cleavage caused the opening of the cohesin ring but not its disassembly, maintaining the binding with SA2 and consequently with CTCF to the viral genome. We suggest that an open cohesin ring structure fails to preserve the linkage between the KSHV latency and lytic control regions essential to keep the latent conformation (Fig 8). Chromosome conformation capture assay revealed that the DNA interactions responsible for KSHV latency genome status were greatly disrupted, already after 3 hours exposure to DTT or puromycin drugs. Taken together, these results strongly suggest that RAD21 cleavage promotes KSHV reactivation affecting the viral episome conformation without influencing cohesin complex binding to KSHV genome. In further support of this model, we show that ectopic expression of the C-terminal RAD21 cleavage product was sufficient to cause KSHV reactivation in BCBLI cells, suggesting that this fragment can function as a dominant negative form of RAD21 and prevent cohesin ring closure. In summary, this study revealed a novel biological function for ER stress-associated RAD21 cleavage as an important in vitro trigger for lytic replication in PEL cells, and provide a chemical model for studying the cellular pathways involved in RAD21-dependent KSHV reactivation. More importantly, these findings suggested a potential molecular explanation for the anti-PEL effects of ER stress inducers that further justify their continued study and use in preventing and treating KSHV-associated tumors.

## Methods

### Cells culture and transfection

BJAB (uninfected B cell lymphoma) cells (ATCC), SLK (uninfected) cells (NIH AIDS reagent program), KSHV positive PEL cells (BCBL1, BC3) (gift of Prof. Yan Yuan, UPENN), and double positive KSHV and EBV infected PEL cells (JSC-1, BC1) (gift of Prof. Yan Yuan, UPENN) were grown in RPMI medium (Gibco/ThermoFisher) containing 10% heat-inactivated fetal bovine serum (Hyclone, Cat. No. SH30396.03, Lot No. AB10092300) and the antibiotics penicillin and streptomycin (50 U/ml). SLK cells were infected with concentrated KSHV-BAC16 viruses derived from induced iSLK-BAC16 cells as described in [15]. Briefly, SLK cells were seeded at 2x10^5^ cells/well in 6 wells plate 24h prior to infection and then inoculate overnight. After 48 hrs, cells were selected by hygromycin at 200ug/ml for 2 weeks and KSHV episome presence was checked by pulse field gel electrophoresis (PFGE). Derived stable lines were designated as SLK-BAC16 cells and cultured in DMEM (Gibco BRL) medium containing 10% heat-inactivated fetal bovine serum, the antibiotics penicillin and streptomycin (50 U/ml) and hygromycin at 200ug/ml. For transient transfection experiments, 10×10^6^ of actively growing BCBLI cells were resuspended in 400 µl RPMI media without serum and antibiotics. 30 µg DNA of interest were mixed together with cells and incubated at RT for 10 min. All transfections were carried out with the Gene Pulser Xcell (Bio-Rad) setting at 250 V and 960 µF as external capacitor. The transfected cells were incubated at RT for 10 min post electroporation, and then maintained as described above.

### Antibodies and reagents

The following antibodies were used for Western blotting: rabbit polyclonal anti-RAD21 (Abcam ab992), rabbit polyclonal anti-PARP1 (Enzo), rabbit polyclonal anti-SMC1 and anti-SMC3 (Bethyl), mouse anti-ORF45 (kindly provided by Prof. Yan Yuan), rabbit polyclonal anti-GFP (Takara), rabbit anti-BiP (Cell Signaling), mouse monoclonal anti-actin-HRP (Sigma), rabbit anti-GAPDH (Cell Signaling), rabbit anti-H3 (abcam) and mouse anti-ORF50 (kindly provided by Prof. Erle Robertson). The following antibodies were used for ChIP assays: anti-IgG (Santa Cruz Biotechnology), anti-CTCF (Millipore), anti-RAD21 (Abcam), anti-RNA polymerase II CTD repeat YSPTSPS (phospho S5) and (phospho S2) from Abcam. Emetine, Puromycin, Pactamycin, Anisomycin, Cycloheximide and Dithiothreitol (DTT) were purchased from Sigma-Aldrich and used at a concentration of 10 ug/ml, 75 ug/ml, 0.2 ug/ml, 10 ug/ml, 100 ug/ml and 5mM, respectively. Subtilase cytotoxin (SubAB) derived from Shiga-toxigenic Escherichia coli (STEC) was kindy provided by Dr. James C. Paton and Adrienne W. Paton at the University of Adelaide,and used at a concentration of 0.1 ug/ml. The Pan-Caspase Inhibitor Z-VAD-FMK (ALX-260-020), the Caspase 9 inhibitor (ALX-260-079), the ER stress inhibitor Salubrinal (ALX-270-428) were purchased from Enzo and used at a final concentration of 20 uM. The Caspase 8 inhibitor (SCP0094) was purchased from Sigma and used at a final concentration of 20 uM. The Calpain inhibitor I was purchased from Cayman chemical and used at a final concentration of 50 uM.

### Western blotting analysis

Western blotting and preparation of samples for SDS-PAGE were performed as described previously [29]. Briefly, proteins were resolved in 8–16% Novex Tris-Glycine gels (Invitrogen), and then transferred onto a PVDF membrane (Millipore), where they were probed with specific antibodies in conjunction with HRP-conjugated secondary antibodies (BioRad) and ECL reagents (Millipore).

### RT-qPCR

RT-PCR was performed as described previously [17].

### Quantification of viral intracellular and virion DNA

The amount of intracellular and virion KSHV DNA was determined by quantitative PCR (qPCR) analysis using primers specific for KSHV ORF50 promoter region as described previously [16].

### Chromatin immunoprecipitation (ChIP)

ChIP assays were performed using the antibodies listed above. Primers for ChIP assays were used as described previously [16]. PCR data were normalized to input values that were quantified in parallel for each experiment.

### Immunoprecipitation (IP)

BCBLI cells were lysed in 100 µL/10^6^ cells of ice cold IP lysis buffer (20mM Tris pH 7.6, 100 mM NaCl, 0.1% NP- 40, and protease inhibitor (Sigma)), incubated for 10 min on ice, then homogenized using 30 strokes of the pestle of a tight-fitting Dounce and centrifuged at 12000 RPM for 10 min at 4°C. The supernatant was precleared by adding 50 ul of protein A sepharose beads (Thermo Scientific) per 1ml of cell lysate for 2 hours at 4°C on rotation. Finally, 500 ug of proteins were diluted in 1 ml of lysis buffer and precipitated using 2 ul of anti-rabbit SMC1 (Bethyl) or anti-rabbit RAD21 (Abcam) O/N at 4°C on rotation. The immunocomplexes were captured by adding 50 ul of protein A sepharose beads to the mixture for 2 hours at 4°C on rotation, then the beads were washed 3 times with 1 ml of lysis buffer, resuspended in 50 ul of 2x Laemmli buffer and boiled for 15 min. SDS-PAGE was performed with 25 ul of the supernatant.

### Isolation of cytoplasmic proteins, soluble nuclear and chromatin fraction

Cells were washed twice with cold PBS before resuspending in buffer A (10 mM HEPES, pH 7.9, 10 mM KCl, 1.5 mM MgCl2, 0.34 M sucrose, 10% (v/v) glycerol, 1 mM DTT, 0.1% (v/v) Triton X-100, supplemented with protease inhibitors (Sigma), and incubated on ice for 8 minutes. By centrifuging at 1,300×g for 5 minutes at 4°C nuclei were isolated, washed once with buffer A (depleted of Triton X- 100) and subsequently lysed in buffer B (3 mM EDTA, 0.2 mM EGTA, 1 mM DTT plus supplements as in buffer A) for 30 min on ice. Soluble and insoluble (chromatin) fraction were separated via centrifugation at 1,700×g for 5 minutes at 4°C. Chromatin samples were washed once with buffer B and subsequently resuspended in SDS sample buffer.

### Chromatin conformation capture (3C)

3C experiments were performed as described previously [7]. PCR data were normalized to cellular actin.

### Construction of GFP-tagged C- terminal RAD21 vector

GFP-tagged RAD21 cDNA encoding the carboxyl-terminal (C-RAD21) caspase cleavage product was constructed by PCR amplifying human wild-type RAD21 with the following primers: 5’-GAATCCAAGCTTGCCACCATGTCAGTGGATCCCGTTGAACCA-3’ and 5’-CGCGCCCTCGAGTCCATATAATATGGAACCTTGG-3’ (C-RAD21 encoding amino acids 280–631). The PCR product was then digested with HindIII and XhoI and cloned into the corresponding sites in front of an in-frame eGFP cassette of a pCS2 derivative. The sequence of the construct fused at its carboxyl terminus with GFP was confirmed by automated DNA sequencing.

### Statistical analysis

p-values were calculated by 2-tailed student t-test using Excel (Microsoft, Redmond, WA).

* p*<*0.05, ** p*<*0.01, *** p*<*0.001.

## Supporting information

S1 Fig Effects of ER stress inducers on PEL cells. **(A-C)** RT-qPCR for latent transcripts (LANA, ORF71, ORF72), lytic transcripts (ORF45-50, PAN), and RAD21 transcript relative to cellular actin in JSC1 cells (C), BC1 (C) and BC3 (D) treated with DMSO, 5mM DTT or 75ug/ml Puromycin (Puro) for 6 hours. The data are expressed as fold change of the treated versus untreated (DMSO) cells. **(D)** Immunoblotting of RAD21, PARP, SMC1 and actin in BC3, JSC1 and BC1 cells exposed to 5mM DTT or 75ug/ml Puromycin (Puro) for 6 hours.
(TIF)

S2 Fig Effects of tunicamycin treatment on RAD21 and KSHV lytic gene expression in BCBLI cells. **(A)** Immunoblotting of RAD21, PARP and actin in BCBLI cells exposed to 10 uM tunicamycin for 6, 12, 24, 36 hours. **(B)** RT-qPCR for LANA, ORF50 and PAN transcripts relative to cellular actin in BCBLI cells treated as in (A). The data are expressed as fold change of the treated versus untreated (DMSO) cells.
(TIF)

S3 Fig Quantification of Western blots shown in Fig 3A. **(A)** The relative levels of the RAD21 cleaved form were obtained by densitometric analysis of the ratio of the specific signals to β-actin in Fig 3A. The specific signals were quantified by densitometric analysis using ImageJ free-share software (http://imagej.nih.gov/ij).
(TIF)

S4 Fig Effect of ER stress on the transcriptional activity of KSHV regulatory lytic control region. **(A)** Schematic of KSHV lytic control region with primer positions A-L used for ChIP assays. Red triangles represent CTCF binding sites. **(B-C)** BCBL1 cells treated for 6 hrs with Puro were assayed by ChIP for IgG or (B) RNA Polymerase II phosphoS5 (RNAPII-S5) and (C) RNA Polymerase II phosphoS2 (RNAPII-S2).
(TIF)
