# Correction: Deregulation of KSHV latency conformation by ER-stress and caspase-dependent RAD21-cleavage

**DOI:** 10.1371/journal.ppat.1007027

**Published:** 2018-04-25

**Authors:** Alessandra De Leo, Horng-Shen Chen, Chih-Chi Andrew Hu, Paul M. Lieberman

There is an error in the XML that is causing the 3rd author’s name, Chih-Chi Andrew Hu, to be indexed incorrectly and affects the citation. The name should be indexed as Hu CC. The correct citation is: De Leo A, Chen H-S, Hu CC, Lieberman PM (2017) Deregulation of KSHV latency conformation by ER-stress and caspase-dependent RAD21-cleavage. PLoS Pathog 13(8): e1006596. https://doi.org/10.1371/journal.ppat.1006596
