# Supplementary figures and images for: Deregulation of KSHV latency conformation by ER-stress and caspase-dependent RAD21-cleavage
Source: PLoS Pathog. 2017 Aug 30;13(8):e1006596. doi: 10.1371/journal.ppat.1006596 (PMC5595345; doi:10.1371/journal.ppat.1006596)

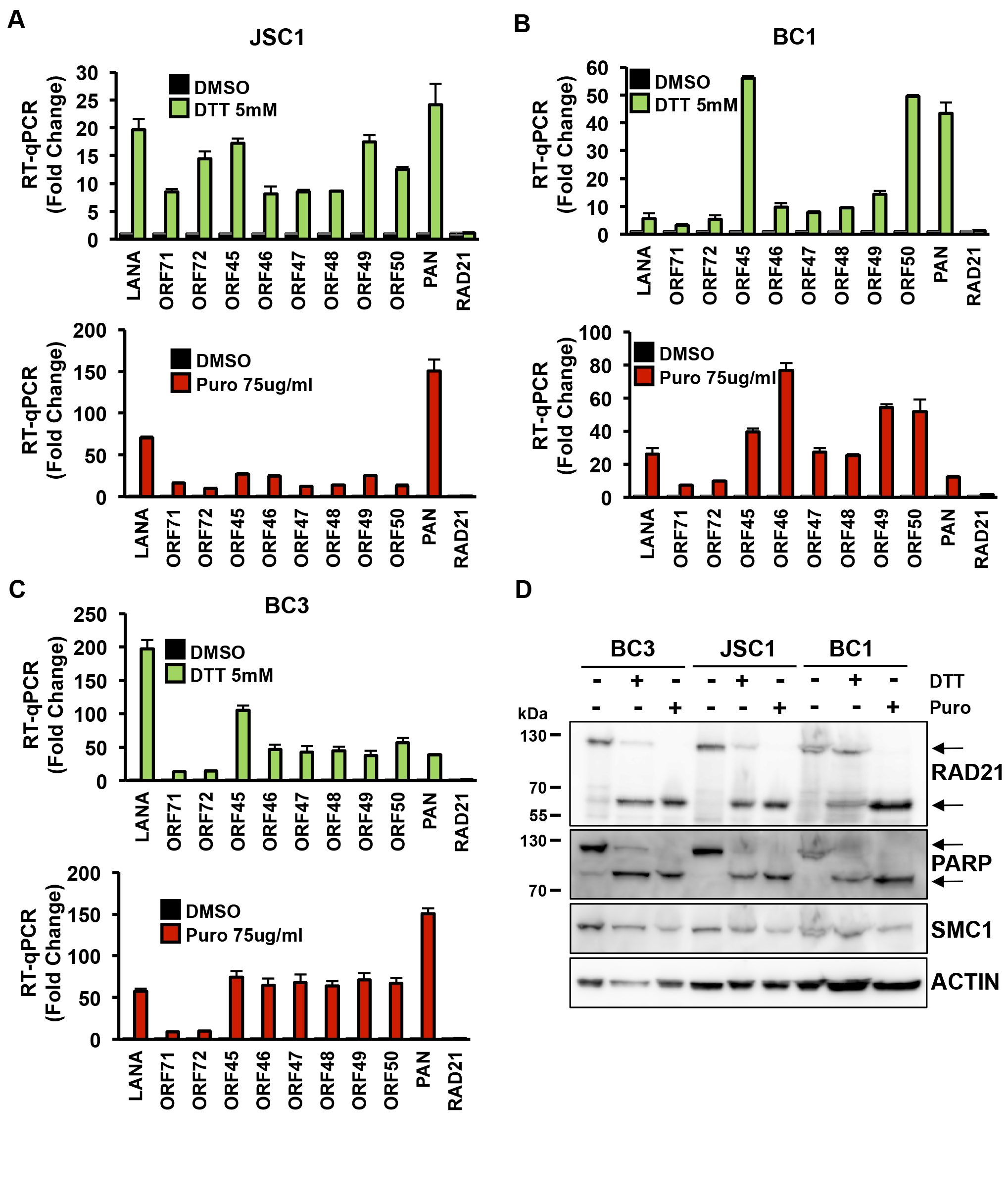

Supplement: S1 Fig — (A-C) RT-qPCR for latent transcripts (LANA, ORF71, ORF72), lytic transcripts (ORF45-50, PAN), and RAD21 transcript relative to cellular actin in JSC1 cells (C), BC1 (C) and BC3 (D) treated with DMSO, 5mM DTT or 75ug/ml Puromycin (Puro) for 6 hours. The data are expressed as fold change of the treated versus untreated (DMSO) cells. (D) Immunoblotting of RAD21, PARP, SMC1 and actin in BC3, JSC1 and BC1 cells exposed to 5mM DTT or 75ug/ml Puromycin (Puro) for 6 hours. (TIF) [file ppat.1006596.s001.tif]

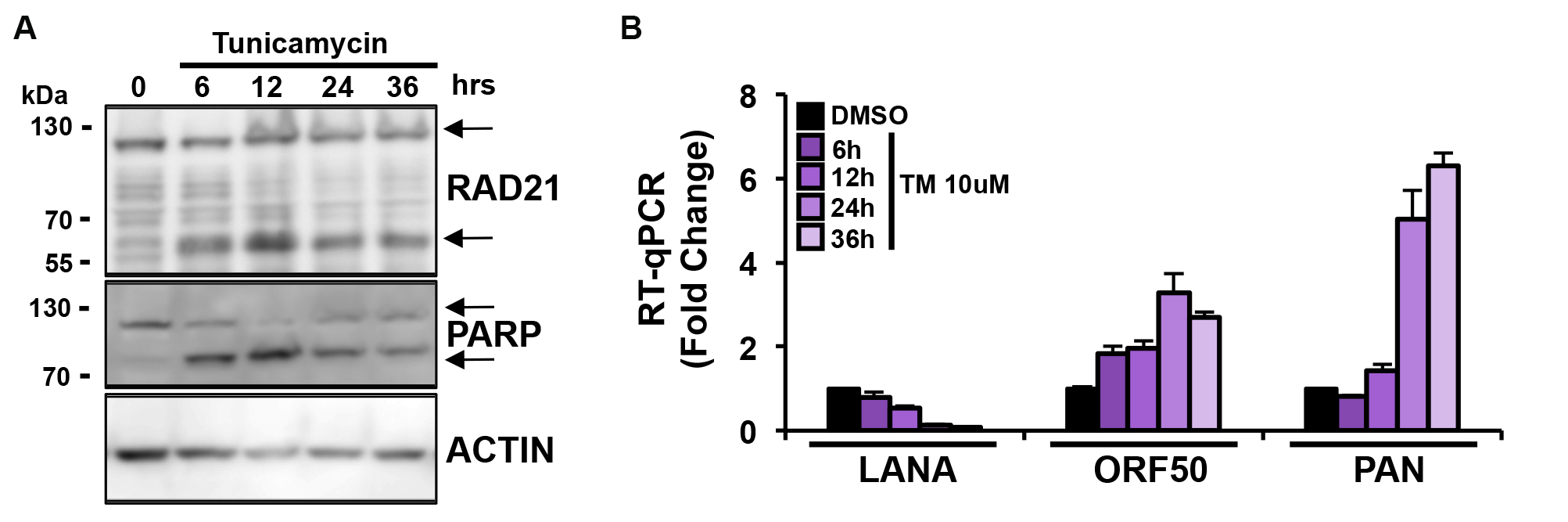

Supplement: S2 Fig — (A) Immunoblotting of RAD21, PARP and actin in BCBLI cells exposed to 10 uM tunicamycin for 6, 12, 24, 36 hours. (B) RT-qPCR for LANA, ORF50 and PAN transcripts relative to cellular actin in BCBLI cells treated as in (A). The data are expressed as fold change of the treated versus untreated (DMSO) cells. (TIF) [file ppat.1006596.s002.tif]

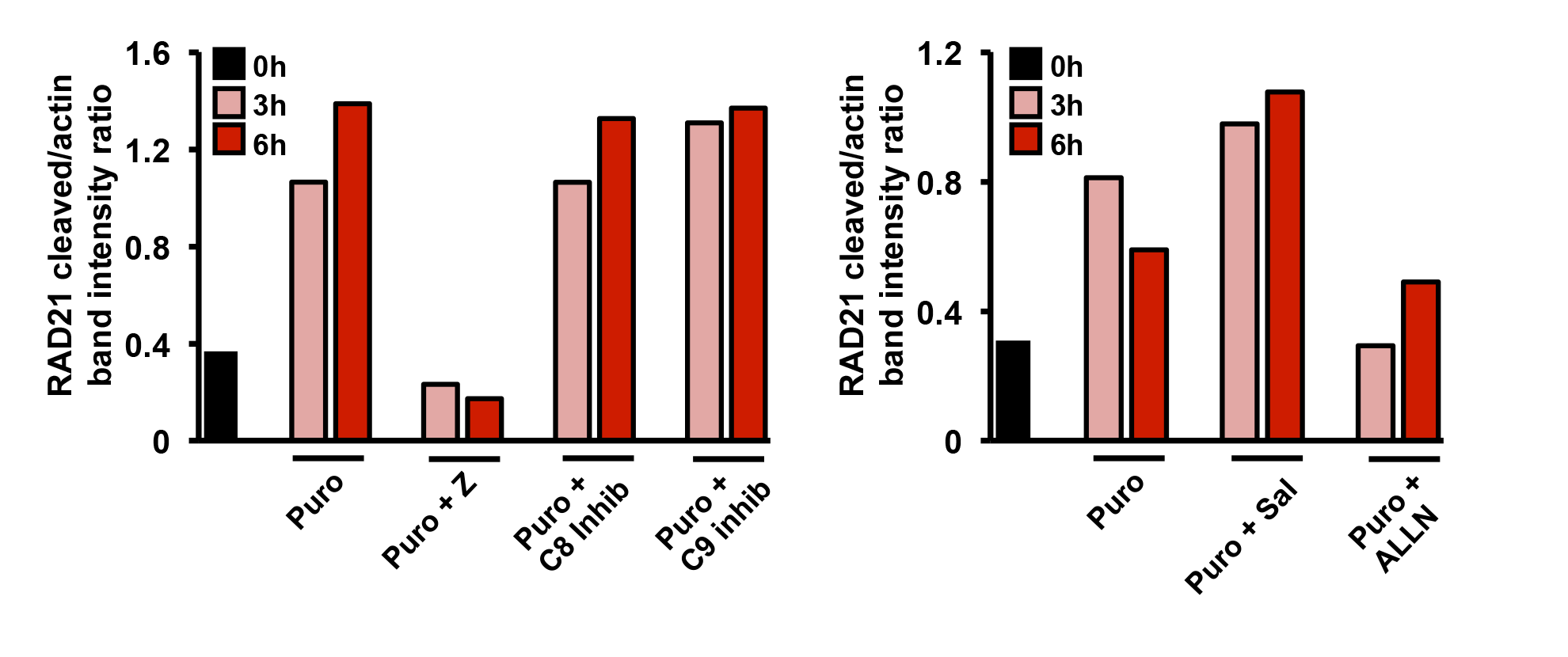

Supplement: S3 Fig — (A) The relative levels of the RAD21 cleaved form were obtained by densitometric analysis of the ratio of the specific signals to β-actin in Fig 3A. The specific signals were quantified by densitometric analysis using ImageJ free-share software (http://imagej.nih.gov/ij). (TIF) [file ppat.1006596.s003.tif]

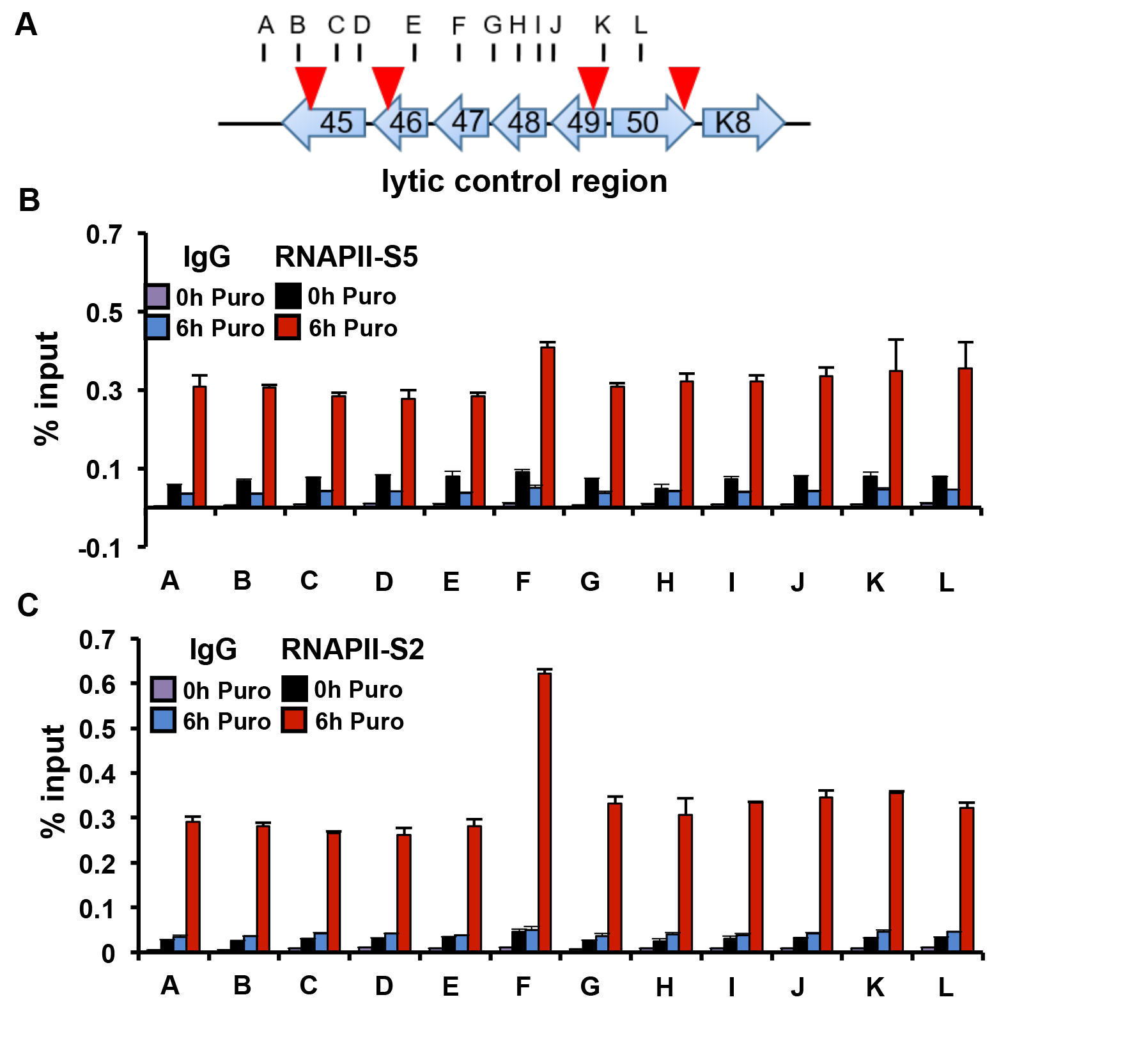

Supplement: S4 Fig — (A) Schematic of KSHV lytic control region with primer positions A-L used for ChIP assays. Red triangles represent CTCF binding sites. (B-C) BCBL1 cells treated for 6 hrs with Puro were assayed by ChIP for IgG or (B) RNA Polymerase II phosphoS5 (RNAPII-S5) and (C) RNA Polymerase II phosphoS2 (RNAPII-S2). (TIF) [file ppat.1006596.s004.tif]
